# An Examination of the Deliberate Practice Framework in Quad Rugby

**DOI:** 10.3389/fpsyg.2019.01734

**Published:** 2019-07-31

**Authors:** Rachel L. Kennedy, Jeffrey T. Fairbrother

**Affiliations:** Motor Behavior Laboratory, Department of Kinesiology, Recreation, and Sport Studies, The University of Tennessee, Knoxville, Knoxville, TN, United States

**Keywords:** deliberate practice, expertise, quad rugby, disability sport, wheelchair rugby

## Abstract

The deliberate practice framework was forwarded to account for the characteristics and developmental experiences of individuals who have acquired exceptional performance in any domain. This framework proposed that experts undergo an extensive acquisition period involving the accumulation of thousands of hours of deliberate practice while overcoming various constraints that serve as functional barriers to the achievement of expertise. Although the deliberate practice framework has been examined in the context of a range of domains, disability sport remains relatively unstudied. The purpose of this study was, therefore, to examine expert disability sport athletes to determine how well their experiences and characteristics were captured by the deliberate practice framework. Quad rugby players were asked to complete a two-part survey to report their recall of the amount of time spent in individual and team practice activities, quad rugby related activities, and daily life activities at the start of their careers and every 2 years since. These activities were then rated with respect to relevance to improving performance, effort and concentration required, and enjoyment of participation. Findings revealed that quad rugby athletes engaged in similar amounts of practice throughout their career to those observed in superior performers across domains, including musicians and expert performers in the able-bodied sport domain (e.g., *M* = 8,309 h at 9–10 year career mark). Contrary to the original deliberate practice framework and some of the subsequent examinations in sport, disability sport athletes did not rate the most relevant and effortful activities as either low or high on enjoyment. The unique constraints imposed on disabled athletes may reduce the likelihood that clear differences will emerge when considering affective responses such as enjoyment.

## Introduction

Superior performance has been documented in a wide range of human performance domains such as music and competitive sport (Ericsson and Smith, 1991). Although many people may attempt to reach such exceptional levels of performance, few have actually reached the highest levels of performance in any given domain (Ericsson, 1996). For example, about 1,700 swimmers competed in the 2016 United States Olympic Trials (Anderson, 2018) to fill a roster of about 50 athletes (USA Swimming, 2016). Less than half of team medaled in individual events. Ericsson et al. (1993) forwarded a general framework to account for the characteristics and developmental experiences of individuals who have acquired exceptional performance in a given domain. This framework proposed that experts undergo an extensive *acquisition period* involving the accumulation of thousands of hours of *deliberate practice* while overcoming various *constraints* that serve as functional barriers to the achievement of expertise.

The presence of an extended acquisition period prior to attaining expertise was originally documented by Simon and Chase (1973) in their examination of international grandmaster chess players. Exceptional performance in this domain appeared to require at least 10 years of intense preparation. Acquisition periods of similar duration (from approximately 6,000 to 10,000 h) have also been observed in international and top-level performers across a variety of other domains including the arts, sciences, and sports (Hayes, 1981; Bloom, 1985; Ericsson and Crutcher, 1990). A more recent review (Baker and Young, 2014) found that the lower end of the range for sport expertise can be as few as about 4,000 h. Additionally, Baker et al. (2003) reported total hours as low as about 2,200. Consistent with the extended acquisition period, several studies have also noted a positive correlation between the age at which an expert begins practice in their given domain and the level of performance ultimately achieved, with international level performers typically showing the youngest starting ages (Ericsson and Crutcher, 1990; Ericsson et al., 1993; Ericsson, 1996). Ericsson and Crutcher (1990) reported that engagement in specific practice activities often begin before the age of six. Subsequent research has indicated that high level performers sometimes sample from a wide range of relevant activities prior to specializing at a later age (e.g., Cote et al., 2003; Hendry and Hodges, 2018).

According to the deliberate practice framework, however, simply practicing for an extended period of time will not automatically result in the achievement of expertise in a domain (Ericsson et al., 1993). Rather, practice activities must fit the criteria for what has been termed deliberate practice. Deliberate practice consists of specifically designed activities focused on accomplishing targeted performance goals and improving skills. Consequently, deliberate practice as originally described does not include paid work, playful interaction, and observing others. Deliberate practice activities are intentionally designed to identify weaknesses and improve aspects of performance through observation of performance results and provision of relevant feedback regarding training goals. The deliberate practice framework places equal emphasis on both the quality and quantity of practice. According to the deliberate practice framework, there is a linear relationship between the amount of deliberate practice accumulated and the level of performance attained. The so-called *monotonic benefits assumption* suggests that a person who has attained the highest level of performance in a domain will also have participated in the largest amount of time in deliberate practice. In fact, according to Ericsson et al. (1993), engagement in deliberate practice is the single most influential contributing factor in the achievement of expert performance in any domain.

During the acquisition period, developing experts must overcome three types of constraints that act to impede progress toward exceptional levels of performance (Ericsson et al., 1993). The *resource constraint* describes the necessity of acquiring access to resources such as teachers, training facilities, and training materials pertinent to the domain. Parents and guardians often play an influential role in overcoming this constraint by providing financial support, transportation, and exposure to the domain. Because of the specialized nature of deliberate practice and the duration of the acquisition period, the resource constraint can place a considerable burden on the available time and money at the disposal of an aspiring expert’s family. The *effort constraint* describes the necessity that an aspiring expert must be willing and capable of devoting the mental and physical effort to maximize each practice session. For many domains, practice can require intense effort that can only be maintained for a short period of time without leading to exhaustion. For example, Ericsson et al. (1993) reported that expert musicians could only engage in about 4 h of deliberate practice activities each day. The *motivational constraint* describes the necessity that an aspiring expert must be motivated to dedicate the time and energy to practice activities that are not inherently enjoyable. Motivation is considered as essential for an aspiring expert to repeatedly produce performances of the highest quality when engaged in deliberate practice.

A strong interest in professional and international sport competition has led several researchers to examine how well the deliberate practice framework describes expertise in sports such as figure skating, wrestling, soccer, field hockey, martial arts, and middle distance running (Hodges and Starkes, 1996; Starkes et al., 1996; Helsen et al., 1998; Hodge and Deakin, 1998; Young and Salmela, 2002). These studies have focused on documenting accumulated hours of deliberate practice and athletes’ ratings of relevance, effort, concentration, and enjoyment of various practice activities as a way of exploring how constraints may have influenced their practice experiences. Although these studies have generally been consistent with Ericsson et al.’s (1993) examination of expert musicians, some notable exceptions have also emerged. For example, Baker et al. (2003) reported total accumulated practice hours ranging from just over 2,200 up to nearly 6,000 for basketball, netball, and field hockey players prior to reaching their respective national teams. Similar findings include the duration of the acquisition period, age at which practice commenced, and accumulated hours of preparation time. In addition, participants in both music and sport studies have rated practice activities most closely matching actual domain performance demands as the most relevant aspects of practice.

Differences between the results found in the domains of music and sport have been related to the way activities are perceived and rated. Specifically, research in the sport domain has not supported Ericsson et al.’s (1993) contention that all deliberate practice activities would be rated high on relevance and effort, but low on inherent enjoyment. For example, in sports such as figure skating, wrestling, martial arts, soccer, field hockey, and middle distance running some practice activities have been rated as both highly relevant and enjoyable (Hodges and Starkes, 1996; Starkes et al., 1996; Helsen et al., 1998; Hodge and Deakin, 1998; Young and Salmela, 2002). Research in the sport domain has also found that participants rate concentration or cognitive effort as distinct from physical effort (e.g., Starkes et al., 1996; Helsen et al., 1998). The divergent findings regarding enjoyment of deliberate practice activities has led some researchers in the sport domain to question if the original deliberate practice framework provides an adequate general description of expertise across domains (e.g., Starkes et al., 1996; Cote, 1999; Macnamara et al., 2016). Hodge and Deakin (1998) noted that “a working definition of deliberate practice has not been realized” and “the framework is in need of revisions if it is to transfer across domains” (p. 277). In response to these observations, work in the sport domain has expanded the original deliberate practice framework to acknowledge that some practice activities can be both highly relevant and enjoyable (Young and Salmela, 2002).

Ericsson et al.’s (1993) deliberate practice framework has been characterized as consistent with the notion that anyone can become an expert by participating in an appropriate amount of deliberate practice (Janelle and Hillman, 2003; Issurin, 2017). Indeed, there are several impressive examples of individuals with disabilities winning Olympic gold medals (Jokl, 1964). Ericsson et al. (1993) suggested that the general nature of the effects of accumulated deliberate practice make it possible for a person with a disability to attain high levels of performance with sufficient practice. They further suggested that the generality of their deliberate practice framework would be supported by research demonstrating practice-driven improvements in performance regardless of the presence of a disability. In short, Ericsson et al. (1993, p. 398) stated that, “…training can compensate for disabilities”. At this point in time, however, little research has been devoted to understanding how the experiences of disability-sport athletes align with the deliberate practice framework. After the initial work examining athletes’ ratings of practice activities, researchers began to focus more on issues related to early specialization and accumulated hours of deliberate practices as factors to distinguish experts from non-experts (Tedesqui et al., 2019). There is, therefore, a need to further examine how well the defining characteristics of the framework align with the experiences of a broader range of sports, including disability sports.

Given Ericsson et al. (1993) claim and the use of individual examples of athletes with disabilities winning Olympic gold medals to support the purported generalizability of the deliberate practice framework, it is somewhat surprising that so little research has been devoted to the relatively large number of individuals with disabilities who regularly participate in high levels of disability sport competitions. If the deliberate practice framework generalizes to the extent that specific training can compensate for a disability in venues such as the Olympics, then it also follows that the framework should describe the characteristics of expert athletes who participate in disability sport competitions such as the Paralympics. On the other hand, because some of the characteristics of experts within the sport domain differ from those proposed in the original deliberate practice framework (e.g., perceived enjoyment of activities), it is also possible that the characteristics of experts in disability sport will differ from those of able-bodied experts. Most research on training in the disability sport domain has either provided a broad overview of training (Hedrick et al., 1988; Watanabe et al., 1992) or has focused only on narrowly defined differences (e.g., brain activity in shooters; Kim et al., 2013), so there is still a need to determine the extent to which the deliberate practice framework captures the characteristics and experiences of disability sport athletes competing at the highest levels of competition.

An examination of expert disability sport athletes might also provide additional insight into the roles that constraints play in the development and maintenance of expertise. In addition to the typical constraints that all aspiring expert athletes face, many disabled athletes must also cope with the constraints imposed by their disability. For example, these athletes may need specialized equipment, for both their sport participation and general use. The availability of training centers and qualified coaches is also likely to be reduced for disability sport athletes compared to their able-bodied counterparts, requiring substantially greater travel demands and/or additional costs. Because daily routines related to personal care and transportation may require a substantial amount of time, some disability sport athletes may face a limitation in accumulating hours of deliberate practice. The examination of expert disability sport athletes will provide insight into theoretical issues related to the generalizability of the deliberate practice framework and the needs of an underserved population in pursuing high-level sports participation. Although the theoretical issues are the primary focus of this study, the practical implications are no less important. According to the International Paralympic Committee, sport participation by people with disabilities can help rehabilitate their physical bodies, integrate them into society, teach independence, and serve as a competitive and recreational outlet (The International Paralympic Committee, 2009).

The purpose of this study was to examine expert disability sport athletes to document their experiences and characteristics. Consistent with previous deliberate practice research, the primary focus of this study was on accumulated hours of deliberate practice and athlete ratings of relevance, effort, concentration, and enjoyment for selected practice and daily life activities. Two research questions were addressed. The first related to how well the deliberate practice framework accurately described the accumulated hours of domain-specific practice for expert disability sport athletes. This study documented the accumulated hours of practice for national and international level quad rugby players to see how closely their practice experiences matched the so-called “10 year rule.” Quad rugby provided a convenient discipline for an initial examination of the deliberate practice framework as it relates to disability sport because it has a national governing body and affords competitive opportunities at national and international levels. Quad rugby athletes therefore fit the framework’s focus on performance at the highest levels. In addition, this study examined whether or not the accumulated amount of practice in quad rugby players was moderated by other factors such as the nature of an athlete’s disability, time spent in non-practice activities, and experiences with other sports (both prior to and during their quad rugby career). The second research question addressed whether expert disability sport athletes rated deliberate practice activities high on relevance and effort, but low on inherent enjoyment as observed by Ericsson et al. (1993) or high on all three dimensions as indicated by subsequent research in the sport domain (e.g., Helsen et al., 1998). In keeping with previous sport domain research, participants also rated activities with respect to the amount of concentration required to distinguish between physical and mental effort (Helsen et al., 1998). This study documented ratings of various practice and non-practice activities that were given by national and international level quad rugby players. Ratings of non-practice activities were included to explore the possibility that constraints unique to disability sport athletes’ activities of daily living might influence their participation in and/or their ratings of deliberate practice activities for quad rugby.

## Materials and Methods

### Participants

Participants in this study were nine men and one woman (*N* = 10), all at least 18 years of age, who had a disability but were otherwise apparently healthy. All participants had the ability to give voluntary and informed consent using a university approved consent document. Participants were recruited from a pool of quad rugby players who competed on a team in the United States Quad Rugby Association. At the time of data collection, the team had won several national championships and selected members had competed internationally on the United States National Team. Recruiting was accomplished through personal contact. The study was approved by the University of Tennessee, Knoxville Institutional Review Board. All participants gave voluntary informed consent.

### Instrument

This study consisted of a single one-on-one session in which participants were asked to complete a written survey adapted from the one used by Young and Salmela (2002). Accommodations were available for any participants who could not complete the survey as designed (e.g., those with difficulty writing due to their disability). This accommodation was provided by the investigator. Prior to conducting the survey, the coach verified that all potential participants had the cognitive capacity to answer the questions on the survey, although many required help with writing.

Part I of the survey requested biographical information concerning the age when practice was first initiated, the highest level attained in quad rugby, success in competitions, and the nature of the participant’s disability. Participants estimated the number of minutes devoted to various practice and life activities during a “typical week” for 2-year intervals since beginning their quad rugby career. Participants were allowed to refer to any personal training logs they brought with them. If participants had competitive experience in other sports, they were asked to provide information for those sports as well.

Part II of the survey required participants to rate various practice and daily life activities on four dimensions related to the deliberate practice framework: relevance to improving quad rugby performance; physical effort required; inherent enjoyment; and concentration (i.e., mental effort) required. Each activity was rated with respect to the four dimensions using a scale of 0 to 10, with 0 being low and 10 being high. When rating activities for relevance participants were instructed that a 0 implied no relevance and a 10 meant the activity was the most relevant for improving quad rugby performance. For effort, a 0 implied no effort and a 10 meant extremely effortful. For enjoyment, a 0 implied the activity was not at all enjoyable and a 10 meant the activity was very enjoyable. For concentration, 10 implied that the activity required no concentration and a 10 implied very high levels of concentration. Activities were separated into four categories: individual practice activities, team practice activities, other quad rugby related activities, and daily life activities as seen in Table 1. Definitions for all activities were available to participants as they completed their ratings.

**TABLE 1:** Taxonomy of various activities related to individual practice, team practice, quad rugby related, and daily life.

**Individual practice**	**Team practice**	**Quad rugby related**	**Daily life**
Cardiovascular	Cardiovascular	Conversing about quad rugby	Active leisure
Flexibility	Flexibility	Coaching quad rugby	Non-active leisure
Game video analysis	Game video analysis	Diet planning	Snacking
Mental training	Mental training	Physiotherapy	Sleeping
Technique	Technique	Reading about quad rugby	Work/study
Weight training	Weight training	Training journal	House duties
Working with a coach	Working with a coach	Watching quad rugby	Transportation
			Body care

### Procedures

The investigator had previously met the members of the team after contacting the coach and visiting the practice facility. During this visit, the investigator introduced herself, spoke individually with several team members, presented a broad overview of the proposed research study, and discussed logistical issues with the coach. For recruiting purposes, the coach provided the investigator with contact information for those team members who expressed interest in participating in the study. During initial contact with potential participants, whether in- person or via phone or electronic mail, the investigator scheduled a time to meet in-person to administer the informed consent and survey. During initial contact, potential participants were given a brief description of the study using the information on the informed consent form and were asked to bring training logs to the meeting if they had them.

During the survey administration meeting, all participants provided voluntary informed consent prior to beginning the survey. Part I and Part II of the survey were administered during the same session in a setting that was mutually agreed upon by both the participant and the investigator. For each part of the survey, verbal and written instructions were provided. The investigator was available throughout the entire administration period and participants had the opportunity to ask questions at any time. Each survey took from 45 to 150 min to complete, depending upon the length of the participant’s career and the nature of any accommodations needed.

### Data Treatment and Analysis

Part I data were treated consistent with previous deliberate practice research. Biographical information was summarized and accumulated amount of practice was calculated as a function of the number of consecutive years involved in quad rugby (Ericsson et al., 1993; Hodges and Starkes, 1996; Helsen et al., 1998). For each athlete, accumulated practice hours were calculated for each 2-year interval by multiplying their total hours per week by the total number of weeks in each 2-year period adjusted for off-season time (104 weeks minus total number of weeks off in 2-year period). Results for each athlete were used to plot data of accumulated hours of practice by years of participation in 2-year intervals, which were then visually inspected to identify the nature of the relationship between these two factors. In addition, disability information and involvement in other activities were summarized and examined to determine if these factors potentially influenced the plotted relationship between accumulated hours and years of participation.

Part II data was examined consistent with previous research (Ericsson et al., 1993; Hodges and Starkes, 1996; Helsen et al., 1998; Young and Salmela, 2002). Ratings for activities were compared to a grand mean for each of the four dimensions rated (relevance, effort, enjoyment, and concentration). Because not all participants rated every activity, overall means were calculated using only the ratings that each participant provided. Comparisons between the ratings for each activity and the dimension grand means were completed using dependent paired *t* -tests. To protect for Type I error inflation a Bonferroni correction was implemented by dividing the original alpha value or 0.05 by the number of activities rated. This resulted in a corrected alpha level set at ≤ 0.002.

## Results

### Part I

#### Biographical Information

The age of quad rugby participants ranged from age 19 to age 40 (*M* = 31.3 year; *SD* = 7.3 year). Reported quad rugby classifications, based upon the classification scale established by the United States Quad Rugby Association, were class 1.0 (*n* = 1); class 2.0 (*n* = 3); class 2.5 (*n* = 2); class 3 (*n* = 2); and class 3.5 (*n* = 2). Lower classification numbers represent greater disability than higher numbers (Gumbert, 2004). Three participants acquired their disability at birth. One participant acquired their disability before age 10. Three participants acquired their disability between ages 15 and 20 and three participants between age 20 and 25.

The age when participants began quad rugby ranged from 15 to 31 (*M* = 22.9 year; *SD* = 4.1 year). The mean age that participants began working with a coach was 23.8 year (*SD* = 4.2 year) and the mean age that participants became engaged in year-round participation was 23.0 year (*SD* = 4.1 year). The mean number of years that participants had engaged in quad rugby ranged from 1 to 18 year (*M* = 8.5 year; *SD* = 6.6 year).

Each participant’s highest level of competition ranged from national level to the international level. All participants were currently training for the national level tournament through the United States Quad Rugby Association. National level was the highest level of competition for six participants, one participant had competed at the international club level, and three participants had competed in the Paralympics.

Half of the participants had been involved competitively in another disability sport prior to or during training for quad rugby. The competitive levels attained in secondary disability sports included: Paralympic swimming and Paralympic track and field event, World Championships in wheelchair racing, U.S. Nationals in track and field event, and Division II college wheelchair basketball. One participant was involved in able-bodied sport at the Division II college level before participating in quad rugby. The other participants had been involved only in the sport of quad rugby.

#### Retrospective Estimates of Time Spent in Practice, Quad Rugby Related, and Daily Life Activities

**FIGURE 1:**
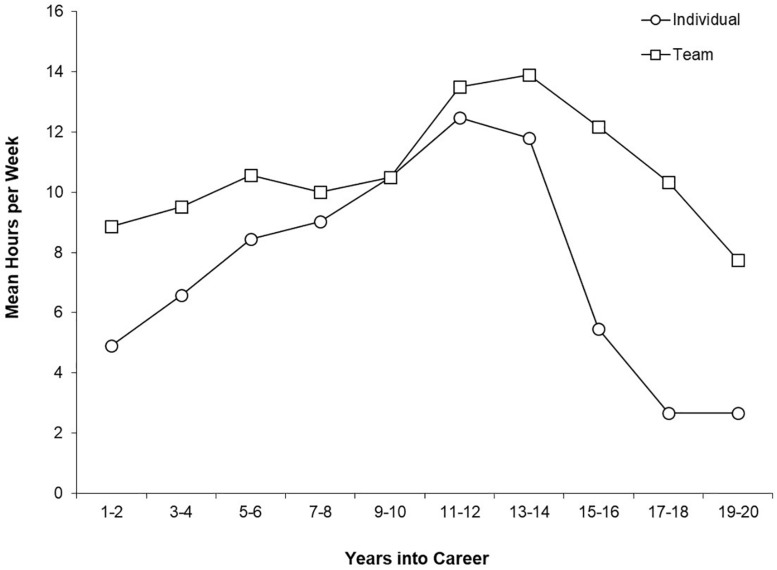
Mean hours per week spent in individual and team practice activities as a function of the number of years into quad rugby career. Hours per week values were based on 10 athletes for Years 1–2; nine athletes for Years 3–4 and Years 5–6; six athletes for Years 7–8; four athletes for Years 9–10, Years 11–12, Years 12–13, Years 13–14, and Years 14–15; and two athletes for Years 17–18 and Years 19–20. The team practice values for two participants in the Years 9–10 and Years 11–12 periods as well as the value for one participant in the Years 13–14 period were excluded because they included hours spent in training camps which did not reflect a “typical week.”

Tables 2, 3 show the descriptive statistics for weekly hours of participation in each of the practice and daily life activities included in the survey. Figure 1 shows the mean hours per week spent in individual and team practice as a function of the number of years into the participants’ quad rugby careers.

**TABLE 2:** Mean hours per week for activities related to individual and team practice.

**Activity**	N	Min	Max	M	SD
**Individual practice**					
Cardiovascular training	10	1.00	9.88	4.28	2.99
Weights	10	0.93	5.75	2.55	1.66
Work with coach	4	0.73	5.82	2.35	2.34
Mental preparation	3	0.83	1.50	1.24	0.36
Technique	5	0.30	1.63	1.16	0.51
Flexibility	8	0.22	1.63	0.84	0.53
Game video analysis	3	0.50	1.00	0.67	0.29
**Team practice**					
Work with coach	10	3.08	11.55	6.37	2.82
Cardiovascular training	10	2.00	10.67	4.57	3.45
Weights	3	1.50	2.00	1.83	0.29
Technique	10	0.25	2.92	1.32	0.77
Mental preparation	7	0.50	2.00	1.21	0.61
Game video analysis	3	1.00	1.40	1.13	0.23
Flexibility	10	0.17	1.47	0.59	0.42

*Data are listed in descending order of mean number of hours per week in each activity category.*

**TABLE 3:** Mean hours per week for activities related to quad rugby and daily life.

**Activity**	N	Min	Max	M	SD
**Quad rugby related**					
Reading about QR	4	1.00	1.83	1.35	0.42
Physiotherapy	8	0.25	1.50	0.94	0.40
Conversing about QR	9	0.03	2.00	0.93	0.58
Organization/preparation	8	0.08	2.25	0.93	0.70
Training journal	3	0.67	1.17	0.92	0.25
Watching QR	5	0.50	1.00	0.85	0.22
Coaching QR	1	0.80	0.80	0.80	0.00
Diet planning	7	0.07	2.42	0.73	0.83
**Daily life**					
Sleep/study	10	44.10	78.55	58.64	11.21
Transportation	10	3.33	160.00	22.71	48.37
Non-active leisure	10	1.00	37.33	14.99	11.43
Snacking	10	1.07	63.00	12.46	18.05
Household duties	9	0.33	21.00	5.87	6.16
Body care	10	1.30	11.67	5.86	3.33
Active leisure	6	0.25	7.00	2.36	2.49

*Data are listed in descending order of mean number of hours per week in each activity category; QR = quad rugby.*

For individual practice activities, all participants engaged in cardiovascular training and weight training, and the majority participated in flexibility training. Five or fewer participants engaged in work with a coach, mental preparation, technique work, and game video analysis. The greatest amount of time was spent in cardiovascular training and the least amount of time was spent in game video analysis. Individual practice comprised 6% of the total time participants devoted to the activity categories represented in the survey. An average of about 5 h per week was devoted to individual practice activities at the start of the career. This amount increased steadily to a peak of about 12 h per week at 11–12 years into the career. After that, the average amount of time decreased dramatically. Because of different career lengths, the number of participants reporting decreased as career length increased.

**FIGURE 2:**
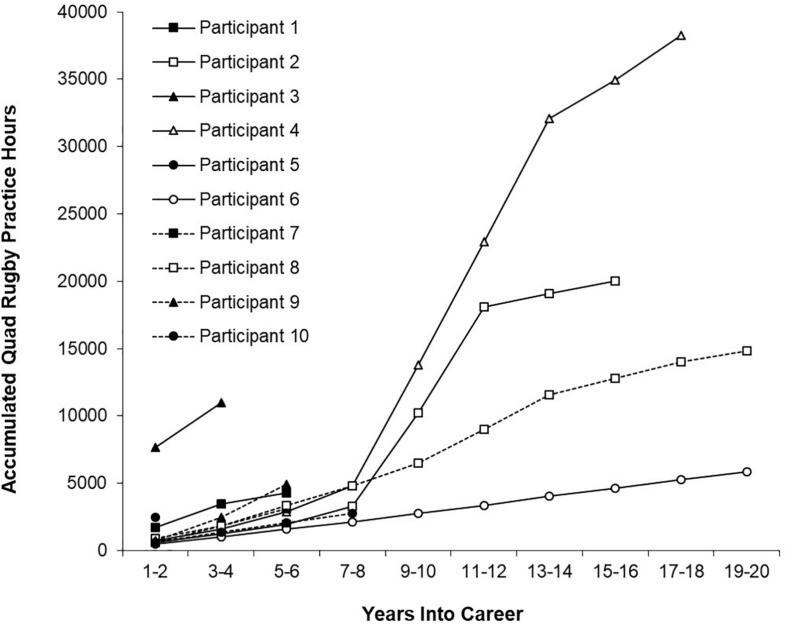
Accumulated hours of practice as a function of the number of years into quad rugby career.

For team practice activities, all participants engaged in work with a coach, cardiovascular training, technique work, and flexibility training. The majority also engaged in mental training. Only three participants engaged in weight training and game video analysis. The greatest amount of time was spent in work with a coach and the least amount of time was spent in flexibility training. Team practice comprised 12% of the total time participants devoted to the activity categories represented in the survey. Figure 2 shows the mean hours per week spent in team and individual practice as a function of the number of years into the participants’ quad rugby careers. An average of about 9 h per week was devoted to team practice activities at the start of the career. This amount increased steadily to a peak of about 14 h per week at 13–14 years into the career. After that, the average amount of time decreased dramatically. Because of different career lengths, the number of participants reporting decreased as career length increased. None of the participants with careers that extended past the 13–14 year point reported additional increases in either individual or team activities beyond that time. Reported weekly time spent in practice activities varied greatly across participants. For the beginning of their careers, reports ranged from 0 to 14 h/week for individual practice activities and from 6 to 16 h/week for team practice activities. By the 13–14 year point, reports ranged from 3 to 27 h/week for individual activities and from 5 to 18 h for team activities.

For quad rugby related activities, a majority of participants engaged in physiotherapy, conversing about quad rugby, organization/preparation, and diet planning. Five or fewer participants engaged in watching quad rugby, reading about quad rugby, and training journals. Only one participant had engaged in coaching quad rugby. The greatest amount of time was spent in reading about quad rugby and the least amount of time was spent in diet planning. Quad rugby related activities comprised 3% of the total time participants devoted to the activity categories represented in the survey. None of the participants engaged in all of the quad rugby related activities.

For daily life activities, all participants engaged in sleep/study, transportation, non-active leisure, snacking, and body care. A majority engaged in household duties and active leisure. The greatest amount of time was spent in sleep and study and the least amount of time was spent in active leisure. Daily life activities comprised 79% of the total time participants devoted to the activity categories represented in the survey.

Figure 2 shows mean accumulated hours of practice in 2-year periods for each participant’s quad rugby careers. At the start of their careers (i.e., the first 2-year period), participants had accumulated from 513 to 1741 h. The four participants that reported for the 9–10 year period had accumulated from 2,744 to 13,768 h, which equated to a yearly average across the 10 years that ranged from 137 to 688 h/year.

Participation in secondary disability sports during their quad rugby career was reported by five participants. Time in secondary disability sport practice at beginning of quad rugby career (*M* = 938 h/year) generally decreased throughout the career (*M* = 600 h/year at 12 year into career), while time in quad rugby practice generally increased.

### Part II

Tables 4–7 display the means and grand means for individual and team practice activities, quad rugby related activities, and daily life activities related to the dimensions of relevance, effort, enjoyment, and concentration.

**TABLE 4:** Ratings for individual practice activities.

**Individual**	N	**Relevance**	**Effort**	**Enjoyment**	**Concentration**
					
		M (GM)	M (GM)	M (GM)	M (GM)
Cardiovascular	10	10.00^*^ (7.65)	9.40^*^ (6.00)	5.00 (6.23)	7.10 (5.86)
Flexibility	10	7.40 (7.65)	4.10 (6.00)	4.60 (6.23)	3.00^*^ (5.86)
Game analysis	6	6.33 (7.62)	4.86 (6.63)	4.43 (6.56)	7.00 (6.41)
Mental preparation	7	8.57 (7.69)	5.29 (6.63)	7.29 (6.56)	9.57^*^ (6.41)
Technique	10	9.50^*^ (7.65)	8.60^*^ (6.00)	7.30 (6.23)	8.70^*^ (5.86)
Weights	10	8.50 (7.65)	8.60 (6.00)	6.10 (6.23)	6.60 (5.86)
Work with coach	8	9.36^*^ (7.66)	6.38 (6.09)	6.75 (6.13)	8.88^*^ (5.95)

0 = low; 10 = high; *GM* = grand mean. *N* = number of responding participants for each pair wise comparison. ^*^p ≤ 0.002.

**TABLE 5:** Ratings for team practice activities.

**Team practice**	N	**Relevance**	**Effort**	**Enjoyment**	**Concentration**
					
		M (GM)	M (GM)	M (GM)	M (GM)
Cardiovascular	10	10.00^*^ (7.65)	9.00^*^ (6.00)	6.20 (6.23)	7.20 (5.86)
Flexibility	9	7.33 (7.60)	4.67 (5.85)	4.67 (6.20)	3.22^*^ (5.85)
Game analysis	4	7.50 (7.75)	5.75 (7.15)	6.50 (7.17)	7.25 (7.08)
Mental preparation	6	7.17 (7.48)	4.67 (6.56)	5.17 (6.79)	7.50 (6.53)
Technique	10	9.10^*^ (7.65)	7.90^*^ (6.00)	6.40 (6.23)	8.00 (5.86)
Weights	4	8.00 (7.67)	8.75 (6.38)	6.25 (6.92)	7.25 (6.47)
Work with coach	10	9.80^*^ (7.65)	6.40 (6.00)	7.70 (6.23)	8.90^*^ (5.86)

0 = low; 10 = high; *GM* = grand mean. *N* = number of responding participants for each pair wise comparison. ^*^p ≤ 0.002.

**TABLE 6:** Ratings for quad rugby related activities.

**Quad rugby related**	N	**Relevance**	**Effort**	**Enjoyment**	**Concentration**
					
		M (GM)	M (GM)	M (GM)	M (GM)
Conversing about QR	10	7.30 (7.65)	5.80 (6.00)	6.70 (6.23)	5.70 (5.86)
Coaching QR	3	9.33 (7.67)	8.33 (6.78)	9.00 (6.97)	9.33 (6.95)
Diet planning	8	8.13 (7.52)	8.25 (6.38)	5.00 (6.44)	7.63 (6.22)
Physiotherapy	4	6.75 (7.53)	6.00 (6.27)	8.50 (6.44)	3.25 (6.33)
Reading QR material	4	5.75 (7.76)	5.25 (7.02)	6.50 (7.04)	5.00 (6.65)
Training journal	3	8.33 (7.81)	7.00 (7.06)	7.67 (7.27)	6.67 (6.90)
Organization/preparation	7	8.71 (7.70)	7.29 (6.07)	5.43 (6.40)	6.14 (5.83)
Watching QR	5	9.20 (7.94)	7.40 (6.71)	6.80 (6.65)	7.00 (6.38)

0 = low; 10 = high; *GM* = grand mean. *N* = number of responding participants for each pair wise comparison; QR = quad rugby. ^*^p ≤ 0.002.

**TABLE 7:** Ratings for daily life activities.

**Daily Life**	N	**Relevance**	**Effort**	**Enjoyment**	**Concentration**
					
		M (GM)	M (GM)	M (GM)	M (GM)
Active leisure	9	6.33 (7.65)	5.22 (5.84)	8.22 (6.16)	4.44 (5.66)
Non-active leisure	10	3.80 (7.65)	2.60 (6.00)	9.30^*^ (6.23)	1.90^*^ (5.86)
Snacking	10	7.60 (7.65)	4.70 (6.00)	8.20 (6.23)	3.40 (5.86)
Sleeping	10	8.80 (7.65)	3.70 (6.00)	9.40^*^ (6.23)	4.10 (5.86)
Work	9	6.78 (7.79)	6.56 (6.15)	4.89 (6.31)	6.67 (5.97)
Household duties	9	1.67^*^ (7.64)	4.56 (6.28)	3.89 (6.29)	3.78 (6.12)
Transportation	10	5.20 (7.65)	4.50 (6.00)	4.40 (6.23)	5.10 (5.86)
Body care	10	7.70 (7.65)	5.90 (6.00)	5.10 (6.23)	5.10 (5.86)

0 = low; 10 = high; *GM* = grand mean. *N* = number of responding participants for each pair wise comparison; QR = quad rugby. ^*^p ≤ 0.002.

For individual practice activities, the means in the relevance dimension were significantly higher than the corresponding grand means for three practice activities – cardiovascular training, *t*(9) = 12.39, *p* < 0.001, technique work, *t*(9) = 8.68, *p* < 0.001, and work with a coach, *t*(7) = 6.47, *p* < 0.001. Means in the effort dimension were also higher than corresponding grand means for two activities – cardiovascular training, *t*(9) = 6.99, *p* < 0.001, and technique work, *t*(9) = 6.80, *p* < 0.001. The mean in the concentration dimension was significantly lower than the corresponding grand mean for flexibility, *t*(9) = −4.62, *p* = 0.001. In contrast, the mean in concentration for work with coach was significantly higher than the corresponding grand mean, *t*(7) = 7.07, *p* < 0.001. Differences between means and grand means in concentration approached significance for mental preparation, *t*(6) = 5.5, *p* = 0.002, and technique, *t*(9) = 4.49, *p* = 0.002. Enjoyment ratings for individual practice activities were not significantly different from their corresponding grand means (all *p* -values > 0.013).

For team practice activities, the means in the relevance dimension were significantly higher than corresponding grand means for cardiovascular training, *t*(9) = 12.39, *p* < 0.001, technique work, *t*(9) = 4.89, *p* = 0.001, and work with a coach, *t*(9) = 14.21, *p* < 0.001. Means in effort were significantly higher than corresponding grand means for cardiovascular training, *t*(9) = 7.75, *p* < 0.001, and technique work, *t*(9) = 4.62, *p* = 0.001. The mean in concentration was significantly lower than the corresponding grand mean for flexibility, *t*(8) = −4.89, *p* = 0.001. In contrast, the mean in concentration for work with coach was significantly higher than the corresponding grand mean, *t*(9) = 10.38, *p* < 0.001. The difference between the mean and grand mean in concentration approached significance for technique, *t*(9) = 3.93, *p* = 0.003. Enjoyment ratings for team practice activities were not significantly different from their corresponding grand means (all *p* -values > 0.023).

For quad rugby related activities, ratings of relevance, effort, concentration, and enjoyment were not significantly different from the corresponding grand means (all *p* -values > 0.006).

For daily life activities, the mean in the relevance dimension was significantly lower than the corresponding grand mean for household duties, *t*(8) = −7.29, *p* = 0.001. The means in enjoyment were significantly higher than the corresponding grand means for non-active leisure, *t*(9) = 6.39, *p* < 0.001, and sleeping, *t*(9) = 6.32, *p* < 0.001. The mean in concentration was significantly lower than the corresponding grand mean for non-active leisure, *t*(9) = −5.92, *p* < 0.001. All other comparisons were not significant.

## Discussion

The first purpose of this study was to examine how well the deliberate practice framework accurately described the accumulated hours of domain-specific practice for expert disability sport athletes. Quad rugby participants in this study reported a mean of 8,309 h (*SD* = 4,756 h) of accumulated team and individual practice at 9 to 10 year into their careers. This was consistent with previous findings on accumulated hours necessary for the attainment of superior performance. Quad rugby participants engaged in similar hours at 9 to 10 year into their careers as those reported by Ericsson et al.’s (1993) best violinists at 10 years (*M* = 6,351 h), Hodges and Starkes’ (1996) wrestlers (*M* = 5,865 h) at 10 years, and Helsen et al.’s (1998) soccer (*M* = 6,328 h) and field hockey athletes (6,559 h) at 13 years into their careers. This study found that disability sport athletes were able to participate in similar amounts of training compared to their able-bodied counterparts. In fact, there was no evidence of additional constraints (e.g., increased daily life preparation requirements, reduced availability of practice locations, and reduced availability of equipment) that negatively affected the number of potential hours available for training. This finding seems at odds with the common perception that a physical disability introduces, at the very least, a need for additional time to complete most daily activities, which raises the possibility that participants in this study sacrificed time in other activities that were not included in the survey. Thus, further research is recommended to clarify this issue.

In contrast to previous findings regarding expertise and deliberate practice, quad rugby participants began practicing well into early adulthood, at about 23 years of age. Thus, it was observed that quad rugby participants begin their career dramatically later than musicians and athletes in able-bodied sports. Presumably, age of entry is influenced by variations in the age at which the disability is acquired. Beginning practice ages have been noted at 8 years of age for musicians, 5 years of age for soccer players, and 13 years of age for wrestlers (Ericsson et al., 1993; Hodges and Starkes, 1996; Helsen et al., 1998). The age of entrance results from this study are similar to previous disability sport research by Hedrick et al. (1988) and Watanabe et al. (1992), which found the age of entrance to disability sport to be an adult phenomenon (age of 24 to 26 year). Therefore, this study concurred with an adult phenomenon of entrance age for disability sport participants. Additionally, participants reported beginning quad rugby, working with a coach, and engaging in year-round participation near the same adult age. This finding shows that high-level performance can still be attained with initiation of deliberate practice late in adulthood for disability sport athletes. Despite some differences from previous research, the results were generally consistent with the notion that practice-driven improvements in performance are possible regardless of the presence of a disability (Ericsson et al., 1993). From a practical standpoint, these findings indicate that quad rugby requires an extensive time commitment over a number of years to reach high-level performance despite that later age of entry and so it should be expected that individual career peaks may occur at later ages than those seen in many other similar sports. Presumably, career peaks might also be of shorter duration as athletes begin to experience aging effects.

Quad rugby participants showed an increase in mean practice hours per week from start of career until the time period of 11 to 14 years into career for both individual and team practice. This was followed by a decrease in the mean number of practice hours per week after 14 years in career for individual and team practice. Previous research on soccer and field hockey revealed a steep increase in mean hours per week from 9 to 15 years followed by an asymptote (Helsen et al., 1998). Helsen et al. (1998) found an increase of time in team practice across career and a decrease of time in individual practice which was not observed in the present study. Across their quad rugby careers, half of the participants were engaged in additional hours of practice for secondary disability sports above and beyond their quad rugby commitments. Because time and energy available for participation are key aspects of Ericsson et al.’s (1993) constraints, competitive participation in secondary disability sports may explain why some participants did not report increases in team practice consistent with expectations of the deliberate practice framework.

Team practice was the primary component of deliberate practice activities, accounting for 12% of the time devoted to all surveyed activities each week. Individual practice comprised the smaller proportion, accounting for 6% of the time devoted to surveyed activities. Because quad rugby is a team sport, it may be that specific activities necessary to the sport are most effectively improved in a team training environment. The importance of such practice with others has been discussed in previous research. For example, even in an individual sport such as wrestling, the importance of sparring practice with partners was rated significantly higher than the mean (Hodges and Starkes, 1996). Similarly, research on soccer and field hockey has found that 65% and 53% of practice time was spent in team practice, respectively (Helsen et al., 1998). Although team practice in the current study represented the largest component of deliberate practice, the proportion of overall time was relatively small compared to the findings for soccer and field hockey. Given that all three sports share some tactical similarities, future research is needed to determine if quad rugby coaches should strive to increase the relative amount of time spent in team training. In the present study, the largest amount of time in team practice occurred in cardiovascular training and working with a coach. Technique work and flexibility were engaged in by all participants, but for a smaller quantity of time during team practice. Game video analysis and working with weights as a team were engaged in by the smallest number of participants. Presumably, a sport such as quad rugby would emphasize tactical and skill dimensions during team training. This perspective is at odds, however, with the finding that cardiovascular training comprised the largest portion of time during team training. The present finding may have been idiosyncratic to the team studied or may represent a unique characteristic of disability sport compared to other sports. More research is needed to document the full range of training activities in other quad rugby teams and across other disabled sports to determine which is the case.

The analyses of individual practice activities revealed that the greatest amount of time was spent in cardiovascular training and weights, with all participants engaging in both activities. Half of the participants engaged in technique work individually, revealing that this type of training was accomplished during both team and individual practice. Relatively few participants engaged in individual practice involving game video analysis and mental preparation. Game video analysis and flexibility contributed the least amount of time for both team and individual practice. It is unknown if the relatively low levels of participation in activities such as game video analysis (individually and with the team) and mental preparation were due to limited availability of resources, time, interest, or other possible factors.

The second purpose of this study was to address whether disability sport athletes would rate deliberate practice activities high on relevance and effort, but low on inherent enjoyment as predicted by Ericsson et al. (1993), or if they would rate these activities as high on all three dimensions as indicated by subsequent research in the sport domain (e.g., Helsen et al., 1998). Similar to previous research, high relevance and high concentration were found for three activities in quad rugby athletes. Individual technique work, working with coach individually, and working with coach in team practice, were all rated high on relevance and concentration. Team technique work was also found to have a high rating for relevance and a (marginally significant) high rating for concentration. This finding was similar to research conducted on musicians (Ericsson et al., 1993), wrestlers (Hodges and Starkes, 1996), and martial arts (Hodge and Deakin, 1998), which found a corresponding relationship between high concentration and high relevance. Previous research has found that activities mirroring the demands of the actual performed activity are rated high in relevance. For quad rugby, ratings of high relevance and high effort were seen in four activities including individual cardiovascular, team cardiovascular, individual technique work, and team technique work. Previous studies have reported ratings of high relevance for practicing alone by musicians (Ericsson et al., 1993), technical and tactical practice by soccer and field hockey players (Helsen et al., 1998), and running by middle distance (Young and Salmela, 2002). In the present study, cardiovascular training and technique work were therefore identified as important components of practice that closely simulate the actual performance demands of competitive quad rugby.

Consistent with previous research, working with a coach was rated high in relevance for both individual and team practice (Ericsson et al., 1993; Hodges and Starkes, 1996; Starkes et al., 1996). Previous disability sport research has not addressed the relevance of a coach from the participant’s perspective. In the present study, working with a coach in a team setting also revealed high ratings for concentration as did both team (presumably under the supervision of a coach) and individual technique work. It is possible that the high degree of concentration for team technique work was partly due to participants’ efforts to interpret the coach’s instructions and feedback. For individual technique work, high concentration may be directed toward self-monitoring efforts. These findings extended current understanding of participant’s perception of the coach and supported the notion that interaction with a coach is a relevant and effortful aspect of quad rugby training.

In previous research, high relevance and concentration ratings for working with a coach have been used to indicate that participants can distinguish between the dimensions of (physical) effort and (mental) concentration (e.g., Hodges and Starkes, 1996). The present results supported this idea. Cardiovascular and technique work were rated as high in (physical) effort for individual and team practice while mental preparation was rated high in (mental) concentration.

Consistent with previous research in the sport domain, high ratings for enjoyment were found for the daily life activities of non-active leisure and sleeping (e.g., Helsen et al., 1998; Hodge and Deakin, 1998). In contrast to expectations emerging from the original framework forwarded by Ericsson et al. (1993), the present study did not find any activities that were rated high for relevance and effort but low for enjoyment. In addition, no activities were rated high for relevance and enjoyment as reported in previous research in the able-bodied sport domain. Instead, the present study found none of the activities were rated significantly higher or lower than the grand mean for enjoyment. These neutral ratings for enjoyment raise the question of what motivates disability sport athletes to engage in deliberate practice activities. The International Paralympic Committee (2009) states that disability sport may provide an outlet for social engagement, competition opportunities, and physical rehabilitation. Perhaps one or more of these potential benefits may be what motivates disability sport athletes.

Overall, the findings from this study revealed limitations in both Ericsson et al.’s (1993) deliberate practice framework and the sport modified framework when describing the characteristics and developmental experiences of disability sport athletes. Nevertheless, many of the characteristics described by Ericsson et al.’s (1993) framework were observed in this study, suggesting that it may prove adequate with some revision to account for the present findings that were unique to disability sport (e.g., neutral ratings of enjoyment). Future research that examines the specific constraints and motivations for disability sport athletes and further documents the characteristics associated with their expertise will contribute not only to the theoretical development of the deliberate practice framework, but will also increase critical knowledge that may benefit an underserved population. The present study had a number of limitations given its narrow focus on members of a single team in quad rugby. Although this approach was successful in describing the characteristics and perceptions of a new group of high-level athletes, the findings may be idiosyncratic to the individuals, team, or sport. Future research is needed to expand current understanding of quad rugby and disability sport athletes. Despite the heterogeneity of the sample in some respects, there was a high degree of variation across individual responses. It is uncertain whether differences in some of the measures (e.g., weekly practice hours) were due to differences in disability, expertise level, motivation, or some combination of these factors. The fact that entrance into disability sport is generally an adult phenomenon precludes examinations of early specialization using the approach of the current study. Such examinations will require much larger sample sizes that will allow for identification and sorting by factors such as degree of disability, age of disability, duration of disability prior to entrance into the sport, and age of entrance into the sport. Future research should also continue the search for objective measures to describe the developmental pathways leading to expertise. Retrospective recall has been questioned as a means for obtaining verifiable data (Howard, 2011), but at the present time better alternatives have yet to be widely adopted. For disability sport athletes, self-report approaches might also be vulnerable to more inaccuracy or inconsistency regarding certain topics. For example, some athletes may feel social pressure to under-report the degree to which extra time devoted to daily activities negatively impacts the time available to pursue training.

## Data Availability

The datasets for this manuscript are not publicly available because of requirements by the University of Tennessee, Knoxville Institutional Review Board. Requests to access the datasets should be directed to the corresponding author.

## Ethics Statement

This study was carried out in accordance with the recommendations of the University of Tennessee Institutional Review Board with written informed consent from all subjects. All subjects gave written informed consent. The protocol was approved by the University of Tennessee Institutional Review Board.

## Author Contributions

RK and JF designed the study, analyzed the data, and wrote the manuscript. RK collected all the data.

## Conflict of Interest Statement

The authors declare that the research was conducted in the absence of any commercial or financial relationships that could be construed as a potential conflict of interest.

## References

[B1] Anderson J. (2018). *Live Updates of the 2020 U.S. Olympic Trials Standards: Swimswam*. Available at: https://swimswam.com/live-updates-of-2020-u-s-olympic-trials-standards/ (accessed June 21, 2019).

[B2] Baker J. Cote J. Abernethy B. (2003). Sport-specific practice and the development of expert decision-making in team ball sports. *J. Appl. Sport Psychol.* 15 12–25. 10.1080/10413200305400

[B3] Baker J. Young B. (2014). 20 years later: deliberate practice and the development of expertise in sport. *Int. Rev. Sport and Exerc. Psychol.* 7 135–157. 10.1080/1750984x.2014.896024

[B4] Bloom B. S. (ed.) (1985). *Developing Talent in Young People*. New York, NY: Ballantine Books.

[B5] Boxell R. L. (2009). *An examination of the Deliberate Practice Framework in quad rugby*. Knoxville, TN: University of Tennessee. Ph.D. thesis.

[B6] Cote J. (1999). The influence of the family in the development of talent in sport. *Sports Psychol.* 13 395–417. 10.1123/tsp.13.4.395

[B7] Cote J. Baker J. Abernathy J. (2003). “From play to practice,” in *Expert Performance in Sports*, eds Starkes J. L. Ericsson K. A. (Champaign: Human Kinetics).

[B8] Ericsson K. A. (1996). “Acquisition of expert performance: an introduction to some of the issues,” in *The Road to Excellence: The Acquisition of Expert Performance in The Arts And Sciences, Sports and Games*, ed. Ericsson K. A. (Hillsdale: Erlbaum).

[B9] Ericsson K. A. Crutcher R. J. (1990). “The nature of exceptional performance,” in *Life-Span Development And Behavior*, eds Baltes P. B. Featherman D. L. Lerner R. M. (Hillsdale, NJ: Erlbaum), 187–217.

[B10] Ericsson K. A. Krampe R. T. Tesch-Römer C. (1993). The role of deliberate practice in the acquisition of expert performance. *Psychol. Rev.* 100 363–406. 10.1037//0033-295x.100.3.363

[B11] Ericsson K. A. Smith J. (1991). “Prospects and limits of the empirical study of expertise: an introduction,” in *Toward a General Theory of Expertise*, eds Ericsson K. A. Smith J. (Cambridge: Cambridge University Press), 1–38.

[B12] Gumbert W. (2004). *BlazeSports Wheelchair Rugby Manual*. Available at: from http://www.blazesports.org/wp-content/uploads/2011/02/BSA-Wheelchair-Rugby-Manual.pdf (accessed June 27, 2019).

[B13] Hayes J. R. (1981). *The Complete Problem Solver*. Philadelphia, PA: Franklin Institute Press.

[B14] Hedrick B. N. Morse M. I. Figoni S. F. (1988). Training practices of elite wheelchair roadracers. *Adapt. Phys. Activ. Q.* 5 140–153. 10.1111/sms.12900 28452146

[B15] Helsen W. F. Starkes J. L. Hodges N. J. (1998). Team sports and the theory of deliberate practice. *J. Sport Exerc. Psychol.* 20 13–35.

[B16] Hendry D. T. Hodges N. J. (2018). Early majority engagement pathway best defines transition from youth to adult elite men’s soccer in the UK: a three time-point retrospective and prospective study. *Psychol. Sport Exerc.* 36 81–89. 10.1016/j.psychsport.2018.01.009

[B17] Hodge T. Deakin J. M. (1998). Deliberate practice and expertise in the martial arts: the role of context in motor recall. *J. Sport Exerc. Psychol.* 20 260–279.

[B18] Hodges N. J. Starkes J. L. (1996). Wrestling with the nature of expertise: a sport specific test of Ericsson, Krampe, Tesh-Römer’s (1993) theory of “deliberate practice.” *Int. J. Sport Psychol.* 27 400–424.

[B19] Howard R. W. (2011). Testing the accuracy of the retrospective recall method used in expertise research. *Behav. Res. Methods* 43 931–941. 10.3758/s13428-011-0120-x 21671138

[B20] International Paralympic Committee. (2009). *History of Sport for Persons With a Disability*. Available at: http://www.paralympic.org/release/Main_Sections_Menu/IPC/About_the_IPC/History_of_Sport_for_Persons_with_a_Disability/ (accessed April 15, 2009).

[B21] Issurin V. B. (2017). Evidence-based prerequisites and precursors of athletic talent: a review. *Sports Med.* 47 1993–2010. 10.1007/s40279-017-0740-0 28493064

[B22] Janelle C. M. Hillman C. H. (2003). “Expert performance in sport: current perspectives and critical issues,” in *Expert Performance in Sports*, eds. Starkes J. L. Ericsson K. A. (Champaign: Human Kinetics).

[B23] Jokl E. (1964). *The Scope of Exercise in Rehabilitation*. Springfield, IL: Charles C Thomas.

[B24] Kim W. Lee G. Kim J. Woo M. (2013). A comparison of cortico-cortical communication during air-pistol shooting in elite disabled and non-disabled shooters. *Pers. Individ. Dif.* 54 946–950. 10.1016/j.paid.2013.01.010

[B25] Macnamara B. N. Moreau D. Hambrick D. Z. (2016). The relationship between deliberate practice and performance in sports: a meta-analysis. *Perspect. Psychol. Sci.* 11 333–350. 10.1177/1745691616635591 27217246

[B26] Simon H. A. Chase W. G. (1973). Skill in chess. *Am. Sci.* 61 394–403.

[B27] Starkes J. L. Deakin J. M. Allard F. Hodges N. J. Hayes A. (1996). “Deliberate practice in sports: What is it anyway” in *The road to excellence: The acquisition of expert performance in the arts and sciences, sports and games* Ericsson K. A. (ed.). Hillsdale, NJ: Erlbaum, 81–106.

[B28] Tedesqui R. A. McCardle L. Bartulovic D. Young B. W. (2019). Toward a more critical dialogue for enhancing self-report surveys in sport expertise and deliberate practice research. *Mov. Sport Sci.Sci. Motricité.* 10.1051/sm/2018027 [Epub ahead of print].

[B29] USA Swimming. (2016). *U.S Olympic Swimming Team*. Available at: https://www.usaswimming.org/docs/default-source/national-teamdocuments/rosters/2016-olympic-team-roster.pdf (accessed June 21, 2019).

[B30] Watanabe K. T. Cooper R. A. Vosse A. J. Baldini F. D. Robertson R. N. (1992). Training practices of athletes who participated in the national wheelchair athletic association training camps. *Adapt. Phys. Activ. Q.* 9 249–260. 10.1123/apaq.9.3.249

[B31] Young B. W. Salmela J. H. (2002). Perceptions of training and deliberate practice of middle distance runners. *Int. J. Sport Psychol.* 33 167–181.

